# Electroporation Parameters for Human Cardiomyocyte Ablation In Vitro

**DOI:** 10.3390/jcdd9080240

**Published:** 2022-07-28

**Authors:** Jara M. Baena-Montes, Tony O’Halloran, Cormac Clarke, Kevin Donaghey, Eoghan Dunne, Martin O’Halloran, Leo R. Quinlan

**Affiliations:** 1 Cellular Physiology Research Laboratory, CÚRAM SFI Centre for Research in Medical Devices, School of Medicine, Human Biology Building, National University of Ireland (NUI), H91 W5P7 Galway, Ireland; jaramariabaena.montes@nuigalway.ie; 2 School of Medicine, National University of Ireland (NUI), H91 W5P7 Galway, Ireland; eoghandonncha.dunne@nuigalway.ie; 3 Aurigen Medical, Atlantic Technological University (ATU) Innovation Hub, H91 FD73 Galway, Ireland; tony.ohalloran@aurigenmedical.com (T.O.); cormac.clarke@aurigenmedical.com (C.C.); kevin.donaghey@aurigenmedical.com (K.D.); 4 Translational Medical Device Lab (TMDLab), Lambe Institute of Translational Research, University College Hospital Galway, H91 Y952 Galway, Ireland; martin.ohalloran@nuigalway.ie; 5 Electrical & Electronic Engineering, School of Engineering, National University of Ireland, H91 HX31 Galway, Ireland

**Keywords:** irreversible electroporation, atrial fibrillation, cardiac ablation

## Abstract

Cardiac ablation with irreversible electroporation (IRE) is quickly being established as a modality of choice for atrial fibrillation treatment. While it has not yet been optimised, IRE has the potential to significantly limit collateral damage and improve cell-specific targeting associated with other energy sources. However, more tissue and cell-specific evidence is required to demonstrate the selective threshold parameters for human cells. The aim here is to determine the optimal ablation threshold parameters related to lesion size for human cardiomyocytes in 2D culture. Conventional biphasic pulses of different field strengths and on-times were delivered in a monolayer culture system of human AC16 cardiomyocytes. The dynamics of cell death and lesion dimensions were examined at different time points. Human cardiomyocytes are susceptible to significant electroporation and cell death at a field strength of 750 V/cm or higher with 100 μs pulses. Increasing the IRE on-time from 3 ms to 60 ms reduces the effective field threshold to 250 V/cm. Using very short pulses of 2 μs and 5 μs also causes significant cell death, but only at fields higher than 1000 V/cm. A longer on-time results in more cell death and induced greater lesion area in 2D models. In addition, different forms of cell death are predicted based on the evolution of cell death over time. This study presents important findings on the ability of different IRE parameters to induce human cardiomyocyte cell death. Lesion size can be tuned by appropriate choice of IRE parameters and cardiomyocytes display an upregulation of delayed cell death 24 h after electroporation, which is an important consideration for clinical practice.

## 1. Introduction

Atrial fibrillation (AFib) is the most common category of cardiac arrhythmia reported in humans characterized by sporadic atria hyperexcitation, resulting in dyssynchronous atrial contraction and irregularity in ventricular excitation [1]. AFib is an increasing global health problem as its prevalence is on the rise, with an estimation of 33 million people worldwide diagnosed with AFib in 2020. This figure is expected to double by 2050 [2]. AFib patients typically present with high morbidity and mortality rates because of the severe consequences associated with thromboembolism and stroke [3]. Moreover, in the last 2 years, infection with COVID-19 is reported to be associated with a more frequent occurrence of arrhythmias [4], thus making AFib a global issue and creating a strong demand for efficient and effective ways to deliver appropriate AFib therapy.

Classically, AFib can be divided into paroxysmal, persistent, long-standing persistent, or permanent, depending on the duration of the symptoms. Paroxysmal AFib is typically resolved within 7 days of onset, while persistent AFib is sustained for longer than 7 days. Long-standing persistent AFib lasts more than 12 months. Permanent AF is used when there has been a joint decision by the patient and clinician to cease further attempts to restore or maintain the sinus rhythm [5]. Despite the sub-classifications, the pathophysiology underlying AFib is not yet fully understood. A key component in the maintenance of the AFib arrhythmia is the process of re-entry, which occurs when impulses travel in an abnormal repetitive circuit [6]. This trigger that initiates the arrhythmia is commonly an ectopic firing focus. The first reported focal ectopic firing was found within the pulmonary veins (PVs) in patients with paroxysmal AF; ablation of these ectopic foci has been shown to reduce AFib burden, demonstrating a role for PVs in AFib [7]. Several subsequent studies have supported the role of PVs and cardiac ganglionated plexi in AFib initiation and highlighted them as targets for therapeutic ablation [8,9,10,11].

Catheter-based pulmonary veins isolation (PVI) is now an established treatment option for AFib. Thermal ablative approaches with either radiofrequency (RF) or cooling (cryotherapy) technologies can cause collateral damage to adjacent structures, including the wall of the oesophagus and the phrenic nerve. These issues have resulted in alternative technologies to be considered, such as irreversible electroporation (IRE) [12,13,14]. The delivery of nano- to milli-second electrical pulses, which are minimally thermal in nature, alters cell membrane permeability, leading to cell death. Cardiac ablation with IRE has yielded some positive data, including a selection of human studies [15,16,17,18,19,20,21]. There are several unanswered questions with regard to the fundamentals of IRE ablation as applied to cardiomyocytes. Previous work from our lab and others has established threshold parameters for IRE with cardiac tissue. However, this work has focused on either immature cardiomyocytes or cells from rodent models [22,23].

The goal of the current study is to assess electroporation ablation thresholds in human cardiomyocytes in suspension and 2D culture. This work provides essential insights into the underlying selectivity of IRE and can be quickly translated to clinical applications.

## 2. Materials and Methods

### 2.1. Cell Culture

Human cardiomyocyte cells AC16 (Merck Life Science Limited Vale Road Arklow Y14 EK18 Ireland) were cultured in T75 flasks and passaged with trypsin-EDTA 0.025% (Sigma-Aldrich, Arklow, Ireland) every 2–3 days for maintenance. Cardiomyocytes were cultured in Dulbecco’s Modified Eagle’s Medium/Ham’s Nutrient Mixture F-12 (DMEM/F-12) (Sigma Aldrich) supplemented with 12.5% fetal bovine serum (FBS) (Gibco, Dublin, Ireland), 1% penicillin/streptomycin (Sigma Aldrich) and 2 mM L-glutamine (Sigma Aldrich). For adherent cell experiments, 6 well plates were coated with 0.1 mg/mL of gelatin before cardiomyocytes were seeded with a cell density of 7.5 × 10^5^ cells per well overnight to ensure complete confluence. For cell suspension experiments, 1 × 10^5^ cells were used per cuvette.

### 2.2. Immunocytochemistry and Staining

Cardiomyocytes were fixed with 4% paraformaldehyde for 20 min and blocked with 0.2% bovine serum albumin (in 0.1% Triton-X100) for 1 h at room temperature. Cardiomyocytes were incubated with troponin I, clone 1F23 ZooMAb rabbit monoclonal antibodies (Sigma-Aldrich, ZRB1355) in blocking solution at 4 °C overnight. Primary antibodies were aspirated and incubated with anti-rabbit 488 fluorophore conjugated secondary antibody 1:1000, (Abcam B.V. Kingsfordweg 151, Amsterdam, 1043 GR, Netherlands) and DAPI in blocking solution for 1 h at room temperature. Cells were imaged using an EVOS M7000 microscope system (ThermoFisher, Fisher Scientific Ireland, Carrigaline, Ireland).

### 2.3. Electroporation Protocols

**Figure 1:**
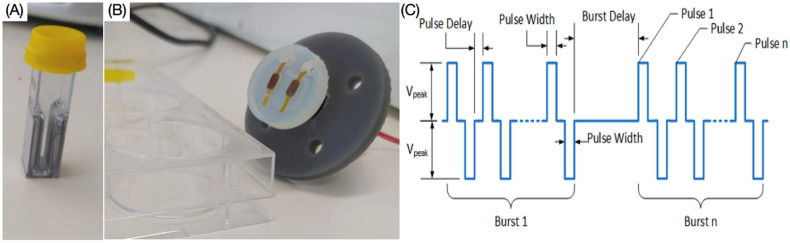
Electroporation equipment for ablation experiments. (**A**) Cuvette with 4 mm gap. (**B**) Adherent cell custom-printed probe. (**C**) Biphasic pulse waveform.

Cardiomyocytes were exposed to a typical irreversible electroporation (IRE) protocol consisting of a 100 μs duration biphasic pulse, delivered in bursts of 5 pulses per burst. Increasing electric fields were generated using the Gemini X2 twin wave electroporation generator (BTX) together with the safety dome (BTX) and the electroporation cuvettes (4 mm gap) for suspension experiments (Figure 1A). In addition, in some experiments, custom electrical field protocols applying 2 μs or 5 μs pulses produced by biphasic research-grade generator were used. To ensure operation with safe limits with this generator, the stimulation pattern was delivered in 100 or 250 bursts, with each burst containing 60 pulses (Figure 1C). For cell suspension studies, field strengths ranged from 0 to 1500 V/cm. For adherent studies, an input voltage of 600 V was chosen as a field strength that was above the ablation threshold and was delivered to the cells directly into the culture well plate using a custom-printed probe for ablation (Figure 1B), with electrode size 3 × 5 mm with a 1 mm fillet, 50 μm in thickness and the edge-edge gap of 5 mm. For the ablation studies, the electrodes were placed 1 mm above the cell layer and covered in DPBS solution. The different ablation protocols and attendant on-times are presented in Table 1.

### 2.4. Live-Dead Assay

Measurements of cell death in the cell suspension experiments were performed by incubating the cells 2 h post-ablation with 3 μM propidium iodide (PI, 30 min, 37 °C) (Sigma-Aldrich). PI is a red-florescent nuclear counterstain, which is commonly used to detect dead cells by binding to their DNA. The PI signal was measured using the Hidex microplate reader (Hidex Sense, Microplate reader, Turku, Finland) at an excitation/emission of 520/620 nm.

Ablation images in adherent cell experiments were assessed by incubation with 3 μM PI and 1.5 μM calcein-AM (30 min, 37 °C) (Sigma-Aldrich) and images were obtained by scanning the wells using the EVOS M7000 Imaging System. Images were analysed using NIH ImageJ to determine the ablation parameters, including area, perimeter, and Feret Max diameter.

### 2.5. Numerical Modelling of 2D Ablation Parameters

A simplified numerical model of the experimental setup was designed in COMSOL Multiphysics^®^ 5.6. The computer-aided design model contained the simplified resin printed probe, the polyimide insulation behind the electrodes and the copper electrodes. In the well, a 100 μm thick layer (34.8 mm in diameter) was used to represent the cell layer and the probe rested ideally on top of this layer. The 100 μm thickness was chosen based on the dimensions of ventricular cardiomyocytes cells given in Vu and Kofidis, (2014) [24]. On top of the cell layer, a layer was drawn for DPBS (7 mm in height). As the conductivity of the cell layer was unknown, the conductivity of DPBS was assigned. The conductivity of DPBS in the model is estimated at 1.2–1.6 S/m [25]. The terminal and ground boundary conditions were assigned to the boundary at the bottom of the electrodes. The applied voltage at the terminal was 600 V. The very high and very low conductivity materials were replaced as boundary conditions in the model at the material interface of the DPBS and cell layers. In this case, the current was not expected to flow through the sides or back of the electrodes, where polyimide insulation was used in the real-world set-up. Additionally, the current was not expected to flow through the low conductivity plastic probe. The electrodes were assumed to have equipotential across the surface. The boundary conditions elsewhere were assumed to be perfectly insulated. The discretisation was then performed for the DPBS layer and the cell layer. A tetrahedral element mesh of approximately 1.88 million elements was used to mesh the model. Similar to previous work by Wasson et al. 2020, the conductivity within the model was assumed constant [26]. A stationary study was run with the electric currents physics in the AC/DC module.

### 2.6. Statistical Analysis

All data were analysed using one-way and two-way ANOVA and multiple comparison tests using IgorPro Ver9. Experiments were repeated for at least three independent experimental blocks with three technical replicates (N = 9). Statistical analyses were performed with a confidence level of α = 0.05.

## 3. Results

### 3.1. Effect of Electric Field Parameters on Cell Death in Suspension Culture

**Figure 2:**
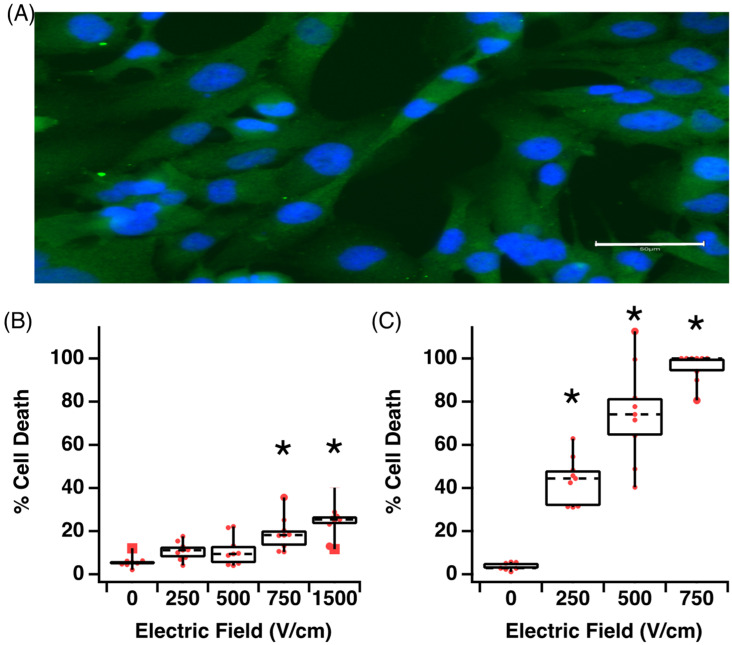
Increasing electrical field strength increases cell death in human cardiomyocytes. (**A**) AC16 cells in culture stained positive for phenotypical markers of human cardiomyocytes, scale bar 50 μm. (**B**) Effect of field strength on cell death with a 3 ms total on-time. (**C**) Effect of field strength on cell death with a 60 ms total on-time. Data are representative of at least 3 independent experiments and plotted as max, min and median. * *p* < 0.05, one-way ANOVA, Dunnett’s post-hoc test.

The AC16 cells stained positive for human troponin I, confirming the human cardiomyocyte phenotype marker (Figure 2A). In the suspension culture, cardiomyocytes were exposed to a standard biphasic waveform consisting of 100 μs negative, followed by 100 μs positive phase with different electric field strengths (250, 500, 750, 1500 V/cm). In addition, the pulse number was varied using either ablation protocol 1 or 2 (Table 1), which is the equivalent of an on-time of 3 ms or 60 ms, respectively. For 3 ms of on-time, cardiomyocyte viability was statistically significantly reduced after exposure to fields greater than 750 V/cm, up to a maximum of 30% cell death at 1500 V/cm (Figure 2B). In addition, 60 ms of on-time resulted in significantly increased cell death at all field strengths (Figure 2C). If we define the optimal ablation threshold as the minimum electric field and on-time resulting in 70% cell death, we can observe that the threshold for cardiomyocytes is 500 V/cm, with an on-time of 60 ms (Figure 2C). Based on this criterion, smaller numbers of pulses are equivalent to a lower on-time and while this may limit the potential risk of collateral damage, it also reduces the effectiveness of the IRE to ablate cardiomyocytes. Longer (60 ms) on-time results in effective cell death across a range of field strength; however, this also increases bubble formation and thermal effects. Hence, higher voltages > 750 V/cm are not included in our analysis (Figure 2C).

**Figure 3:**
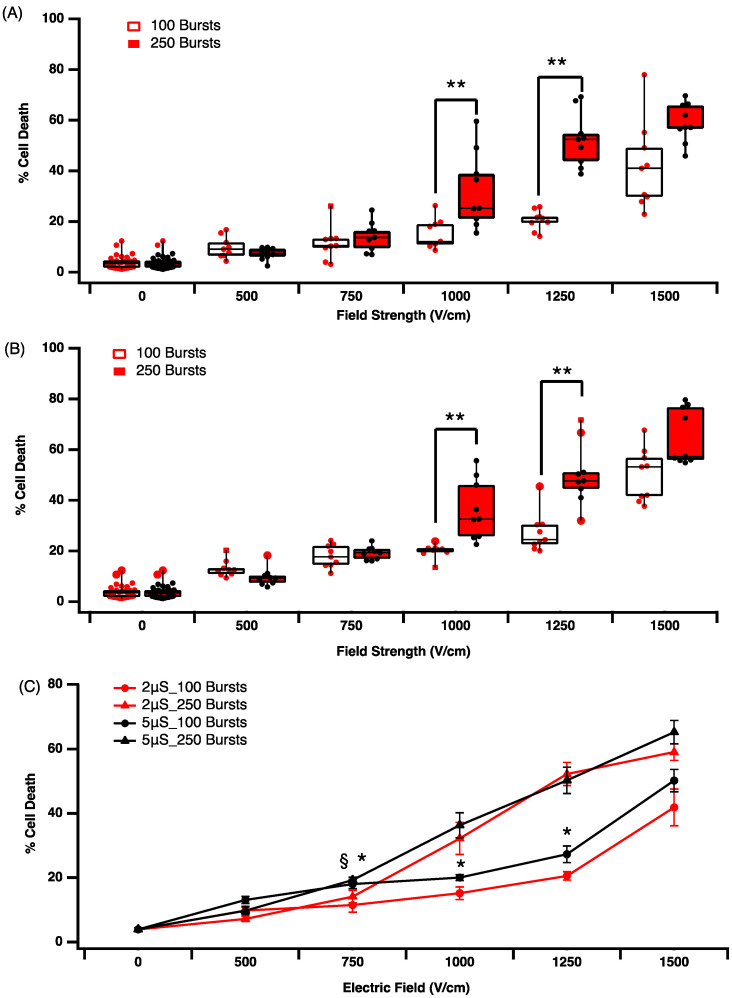
Increasing pulse duration, burst number and field strength reduces cardiomyocyte viability. (**A**) Effect of 2 μs pulse duration on cell survival. (**B**) Effect of 5 μs pulse duration on cell survival. (**C**) Comparison of 2 μs versus 5 μs pulse duration on human cardiomyocyte survival. Data are representative of at least 3 independent experiments and plotted as max, min and median. § *p* < 0.05, * *p* < 0.005, ** *p* < 0.001, one-way ANOVA, Dunnett’s post-hoc test.

The literature suggests that shorter pulse durations in the micro or even nanosecond domain may reduce the rise of hydrolysis and arcing [27]. To examine the effectiveness of short pulse durations on cell death, we compared 2 μs versus 5 μs pulse durations using biphasic waveform for 100 (ablation protocol 3 and 4, Table 1) or 250 bursts (ablation protocol 5 and 6, Table 1) in suspension culture. The application of 2 μs pulses resulted in significant cell death at electric fields of 750 V/cm and above (Figure 3A). In addition, at 1000 V/cm and 1250 V/cm, 250 bursts resulted in significantly (** *p* < 0.005) more cell death than 100 bursts and this differential disappeared at 1500 V/cm. In line with the previous data, increasing pulse duration from 2 μs to 5 μs increases cell death overall (Figure 3B). The difference observed when jumping from 1000 V/cm to 1250 V/cm is also significant (** *p* < 0.005) for 250 bursts and more effective at causing cell death than 100 bursts (Figure 3B). In comparing across treatment groups, a field strength greater than 750 V/cm increases cell death, independent of pulse duration or burst number applied (Figure 3C). However, it is also evident that at higher burst numbers (250 bursts, § *p* < 0.05), there is only a minimal additional effect of pulse duration at voltages greater than 750 V/cm. Using lower burst numbers (100 bursts) at 750, 1000 and 1250 V/cm, we observe statistical differences in percentage cell death, with the 5 μs pulse being more effective than 2 μs pulses (Figure 3C, * *p* < 0.005).

### 3.2. Effect of Time on Cell Death and Lesion Size in a 2D Culture Model

**Figure 4:**
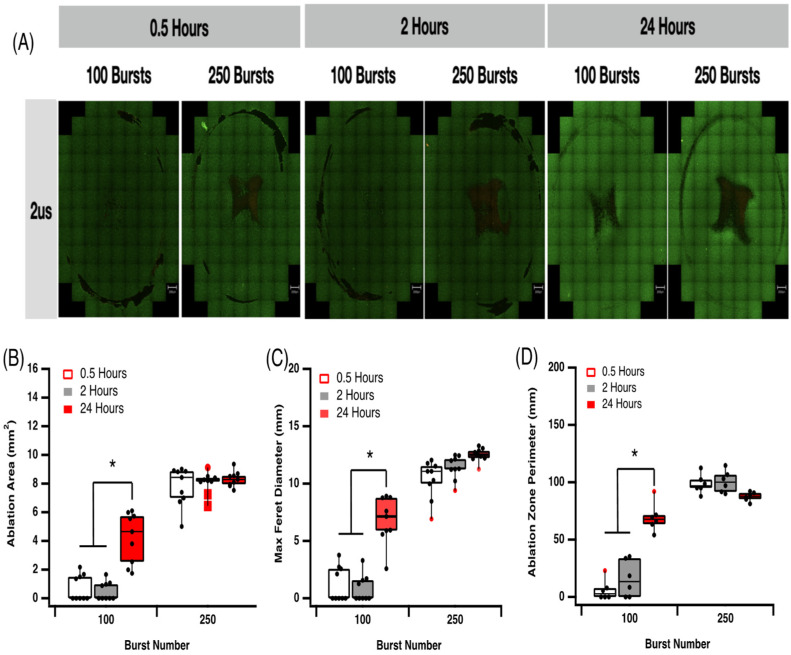
Effect of 2 μs pulses and burst number on cardiomyocyte lesion size. (**A**) Effect of 2 μs pulse duration on cell survival. (**B**) Effect of burst number over time on ablation area. (**C**) Effect of burst number over time on max Feret diameter. (**D**) Effect of burst number over time on ablation zone perimeter. Data are representative of at least 3 independent experiments and plotted as max, min and median. * *p* < 0.05, one-way ANOVA, Dunnett’s post-hoc test.

Cells were grown in wells to near full confluency in this 2D model. Cells were exposed to a lethal ablation threshold established in earlier experiments and live-dead analysis was assessed with propidium iodide (PI) staining at 0.5, 2 and 24 h post electroporation. Exposure to a 2 μs pulse at 600 V/cm with 100 or 250 bursts (ablation protocol 3 and 4, Table 1) results in increasing time-dependent cell death, with 250 bursts always resulting in more cell death at each time point (Figure 4A). The ablation area, max Feret diameter and ablation perimeter (Figure 4B–D) all increase significantly with 250 bursts compared to 100 busts. We found no significant difference over time with higher pulse numbers. However, at 100 busts, we observe a significant increase in all parameters measured 24 h post-ablation. This indicates a possible delay in cell death pathway activation by IRE, which is masked when higher burst numbers are applied. It is most likely that this is due to the activation of apoptosis pathways, which are activated by IRE and take more than 2 h to execute their program (Figure 4B–D).

**Figure 5:**
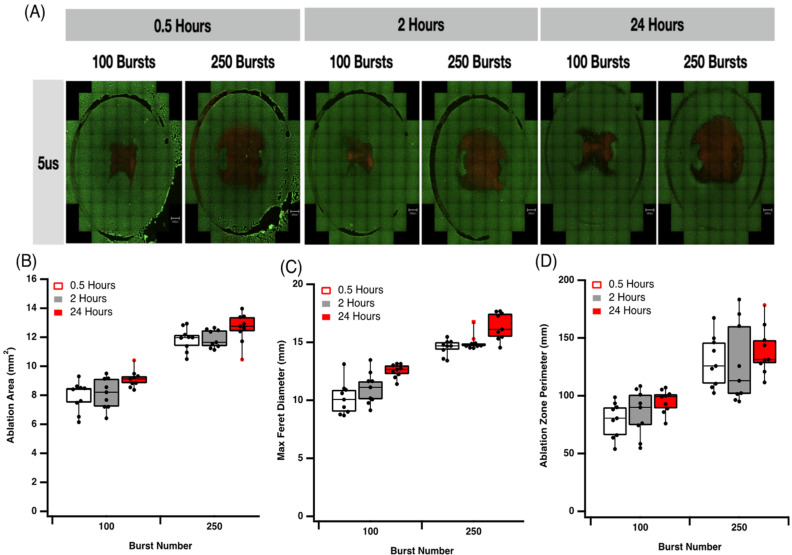
Effect of 5 μs pulses and burst number on cardiomyocyte lesion size. (**A**) Effect of 5 μs pulse duration on cell survival. (**B**) Effect of burst number over time on ablation area. (**C**) Effect of burst number over time on max Feret diameter. (**D**) Effect of burst number over time on ablation zone perimeter. Data are representative of at least 3 independent experiments and plotted as max, min and median.

**Figure 6:**
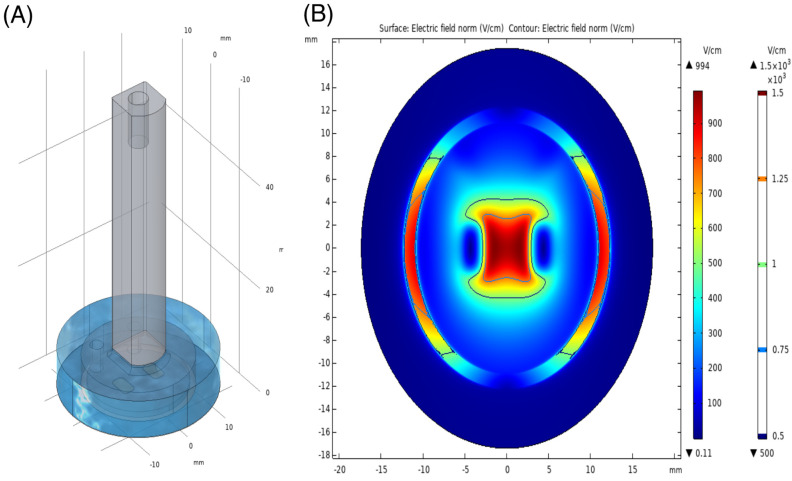
The numerical model (**A**) and the electric field strength plot (**B**) of the 2D electroporation at half the cell layer thickness of 50 μm.

A similar pattern of lesion formation is observed for 5 μs pulses (ablation protocol 5 and 6, Table 1) in our 2D model. Increasing burst number increases cell death, ablation area, max Feret diameter and the ablation perimeter (Figure 5). At the higher field strengths, the time-dependent increase in cell death is not significant, in contrast to that observed at 2 μs. While increasing pulse amplitude (2 μs versus 5 μs) and increasing burst number (100 versus 250) results in a greater ablation area (Figure 5A,B), max Feret diameter (Figure 5C) and ablation zone perimeter (Figure 5D), these data suggest that two different cell death mechanisms are at play. Time-dependent changes are only observed at the lower pulse amplitudes, suggesting that higher intensity electric fields with shorter duration on-times may allow for more controlled cell death. This trend is consistent with the profile of field contours demonstrated in the in-silico model we generated. The ablation zone associated with our IRE treatment was simulated using COMSOL Multiphysics software (Figure 6). Based on our data, we predict that cell death via necrosis occurs in the proximity of the epicentre of the electric field, with a predicted field strength close to 900 V/cm. As the electrical field drops off toward the periphery, other forms of cellular response are anticipated, including apoptosis and reversible electroporation.

## 4. Discussion

Our data clearly demonstrate that human cardiomyocytes are susceptible to damage and cell death from electroporation across a range of pulse parameters, which suggests that there is significant room for optimisation in clinical IRE application. At a cellular level, IRE results in the formation of hydrophilic nanopores in the cell membrane. At a higher intensity, these changes are permanent, having long-lasting/irreversible effects on permeability across the phospholipid membrane [28]. In general, our data show that increasing field strength increases cell death, but importantly, increasing the number of pulses for a given electric field can have significant benefits in terms of ablation. Using a conventional 100 μs biphasic pulse at 500 V/cm with 3 ms on-time (ablation protocol 1) results in approximately 15% cell death. Using the same parameters, increasing only the on-time to 60 ms (ablation protocol 2) results in 75% cell death (Figure 2). Others have shown significant IRE-induced ablation of H9C2 rat cardiomyocytes at field strengths greater than 375 V/cm [29]. More recently, Hunter et al. reported that biphasic pulses at 500 V/cm caused 80% cell death in monolayers of rat ventricular cardiomyocytes [23]. However, in this case, the authors used a single pulse of 10 ms (5 ms for each phase), in stark contrast to our pulse parameters. In a recent review by Sugrue et al., (2018) where they examined 16 eligible clinical IRE cardiac ablation studies, they reported that the pulse duration varied from 20 μs to 6 ms and the number of applied pulses varied between 1 and 200 per procedure [30]. Thus, it is clear that while both monophasic and biphasic pulses can be effective, the advantages or disadvantages of either waveform type, pulse duration or pulse number on lesion formation are unclear and require additional study.

There is increasing interest in applying shorter pulsed electric fields for IRE, using pulses in the nano- and microsecond domains. The theory is that this may further circumvent the limitations of RF- and cryoablation, and also reduce neuro and neuromuscular activation. Ablation with nsPEFs has been successfully used for tumours [31], but there is minimal evidence in cardiac tissue [32]. Here, we show that a short pulse of 2 or 5 μs can be effective in inducing ablation in suspension culture (Figure 3) and that altering pulse length and number can facilitate the control of lesion size in 2D cultures (Figure 4 and Figure 5). This is in line with previous work that showed that at higher field strengths, the percentage of cell damage is greater for mouse cardiomyocytes [23]. When comparing the ablation protocol with the equivalent on-time but different pulse length, we observe some interesting data. Ablation protocols 2 and 4 have the same on-time but pulse lengths of 100 μs versus 2 μs, respectively. At a field strength of 500 V/cm, protocol 2 results in approximately 75% cell death, while the protocol for the same field strength results in approximately only 10% cell death. In addition, 750 V/cm protocol 2 results in approximately 100% cell death, while the protocol for the same field strength results in approximately only 18% cell death. Clearly, pulse duration has a very significant effect on cell death.

Furthermore, these data suggest that there is a time dependence element to cardiomyocyte cell death, and at voltages near the ablation threshold (600 V/cm), significant cell death can emerge 24 h post-ablation. At higher field strengths, cardiomyocyte death is more consistent and is less influenced by the time post-ablation. We show that a fraction of cardiac cells die almost immediately after the treatment, regardless of the electric field. This type of response was also reported by others for cancer cells [31,33].

In recent years, there has been considerable development in catheter design that supports so-called single-shot applications (Farapulse Inc., Menlo Park, CA, USA), or the more geometrically complex Sphere-9 catheters (Affera Inc. Newton, MA, USA), originally designed for RF but are also able to deliver bi-phasic pulsed electric fields. The investigation of optimal IRE parameters to maximise tissue selectively and to control the temporal and spatial aspects of lesions is limited. The acute and chronic assessment of lesion durability and/or lesion expansion after IRE needs further investigation. Our data support the idea that appropriate parameter selection can deliver tissue selectivity far in advance of other modalities. This ultimately will improve safety, reduce collateral damage and reduce procedure times in the clinic. However, to fully realise the clinical potential of IRE, this will require the determination of the optimal IRE parameters, and an important consideration in this regard will be the appropriate monitoring time after the application of IRE for the accurate assessment of cell death. Previous studies have pointed out the significance of observational times when carrying out a histological evaluation of the ablated areas in animal models treated with IRE [34].

Cell death pathway activation has been shown to be dependent on several variables, including field strength and pulse number [35]. Predicting the effect of IRE and driving cell death down a particular pathway may have advantages in the clinic by reducing inflammatory and immunogenic responses [36]. While we did not measure cell death pathway activation, the 24-h delay in the response to some IRE waveforms strongly suggests that apoptosis was activated. This may be beneficial, as it is associated with reduced inflammatory responses. It is likely based on our modelling (Figure 6) that cells at the centre of the field are dying of necrosis, whereas as the field contours reduce, more subtle changes in cell responses are likely. These responses will include the activation of apoptosis and reversible EP from which cells can recover. Our model maps well to our cell death profile in the 2D adherent cell culture model and will provide an important assessment tool for studying changes in field parameters and lesion size. Further studies are required to fully elucidate the effect of conventional IRE on cell death pathways and the tuneable nature of lesion size.

### Limitations

In this study, the 2D monolayer culture of human cardiomyocytes facilitated the testing of IRE parameters, allowing the iterative exploration of pulse protocols. However, while these AC16 cells are a very close representation of cardiac muscle, our model is limited by the lack of 3D geometry, which can be addressed by building appropriate 3D cardiac cellular models. In our in-silico model, we make assumptions about the conductivity of the surrounding fluids and tissues, which may differ substantially from native tissues, and thus affect the electric field thresholds applied. However, we are confident that the relative differences observed comparing the various IRE protocols would be representative of the in vivo scenario. Future studies incorporating a three-dimensional model with more representative conductivities are required to allow full extension of the identified ablation thresholds to the clinic.

## 5. Conclusions

Our results highlight the potential to tune IRE protocols to achieve efficient and selective cell death in cardiac applications. Furthermore, our data highlight the potential to minimise inflammatory responses by selectively inducing cell death through the activation of delayed cell death pathways. We show that the on-time though pulse timing or burst number, rather than absolute field strength, may be a better parameter to tune a more predictable ablation area. These results provide new insights into the field of cardiac IRE but must be explored further in order to be fully extrapolated to clinical outcomes.

## Figures and Tables

**Table 1:** Ablation protocols applied to different models of cardiac cell culture.

Ablation Protocol	Pulse Width (μs)	Pulse Number	Burst Number	On-Time (ms)
1	100	5	10	3
2	100	5	60	60
3	2	60	100	24
4	2	60	250	60
5	5	60	100	30
6	5	60	250	75

## Data Availability

The data underlying this article will be shared upon reasonable request to the corresponding author.
